# Characterization of the lung microbiome and inflammatory cytokine levels in women exposed to environmental risk factors: A pilot study

**DOI:** 10.1002/iid3.825

**Published:** 2023-04-17

**Authors:** Fernando Morales‐González, Juan A. Lira‐Lucio, Ramcés Falfán‐Valencia, José E. Márquez‐García, Edgar Abarca‐Rojano, Alejandra Ramírez‐Venegas, Raúl H. Sansores, Leonor García‐Gómez, Andrea Hernández‐Pérez, Gloria Pérez‐Rubio

**Affiliations:** ^1^ HLA Laboratory, Instituto Nacional de Enfermedades Respiratorias Ismael Cosío Villegas Mexico City Mexico; ^2^ Subdirección de Investigación Biomédica, Instituto Nacional de Enfermedades Respiratorias Ismael Cosío Villegas Mexico City Mexico; ^3^ Sección de Estudios de Posgrado e Investigación, Escuela Superior de Medicina, Instituto Politécnico Nacional Mexico City Mexico; ^4^ Department of Tobacco Smoking and COPD Research Instituto Nacional de Enfermedades Respiratorias Ismael Cosío Villegas Mexico City Mexico; ^5^ Clínica de Enfermedades Respiratorias, Fundación Médica Sur Mexico City Mexico

**Keywords:** dysbiosis, immunity, inflammation, respiratory disease, respiratory microbiome

## Abstract

**Introduction:**

Lung microbiome dysbiosis affects the immune system balance and promotes lung inflammation. We aimed to characterize and compare the lung bacteriome composition and the cytokine profile in women with normal lung function exposed to risk factors for chronic lung diseases (tobacco smoking and biomass‐burning smoke exposure).

**Methods:**

We included women with biomass‐burning smoke exposure (BE, *n* = 11) and current smokers women (TS, *n* = 10). The bacteriome composition was performed in induced sputum, sequencing the 16 rRNA gene. Cytokine levels were measured using enzyme‐linked immunosorbent assay multiplex assay in the supernatant of induced sputum. For quantitative variables, we used medians and minimum and maxim values. For the amplicon sequence variants (ASV) differential abundance testing between groups.

**Results:**

At the taxa level, the phylum Proteobacteria was found in a higher proportion in the TS group concerning BE (*p* = .045); however, after the false discovery rate adjustment, this difference was not retained (*p* = .288). We found a higher concentration of IL‐1β in the TS group than in the BE group (248.6 vs. 177.9 pg/mL, *p* = .010). Women with high biomass‐burning smoke exposure in an hour per day had a positive correlation with the abundance of Bacteroidota (*ρ* = 0.71, *p* = .014) and Fusobacteriota (*ρ* = 0.73, *p* = .011). FEV1/FVC had a positive correlation with an abundance of Bacteroidota, Proteobacteria, and Fusobacteria (*ρ* = 0.74, *p* = .009, *ρ* = 0.85, *p* = .001, and *ρ* = 0.83, *p* = .001, respectively). In tobacco smoking, women had a positive correlation (*ρ* = 0.77, *p* = .009) between cigarettes per day and Firmicutes' abundance.

**Conclusion:**

Compared to biomass‐burning smoke‐exposed women, current smokers have poor lung function and high levels of IL‐1β in sputum. Women with biomass‐burning smoke exposure present an increased abundance of Bacteroidota and Fusobacteriota.

## Graphical abstract

This is the first study that evaluates the bacterial composition in exposed women to known risk factors for lung diseases with normal lung function. The pro‐inflammatory mediators play an essential role in the initiation and maintenance of inflammation.

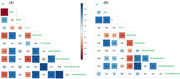

## 1 INTRODUCTION

Inhalation of toxic substances such as smoke from biomass combustion (common in poorly ventilated homes) and tobacco smoking are risk factors for developing respiratory tract diseases (lung cancer, chronic obstructive pulmonary disease [COPD], asthma, and others). The compounds and particles in these noxious gases cause changes in pH, oxygen tension, oxidative stress, pro‐inflammatory conditions, and even dysbiosis of the microbiome in the pulmonary microenvironment.^1^ Five phyla are the most abundant in healthy lungs Firmicutes, Proteobacteria, Bacteroidota, Actinobacteriota, and Fusobacteria.^2^, ^3^, ^4^

Cigarette smoking influences the composition of the lung microbiome,^5^ reporting a positive correlation between the cigarette smoking years and taxa of the Firmicutes phylum (genera *Veillonella* and *Megasphaera*) and the genus *Prevotella*. At the same time, this variable is inversely associated with Proteobacteria (genera *Eikenella* and *Haemophilus*).^6^ Furthermore, smokers have a 20%–30% higher probability of needing antibiotics.^7^ In COPD patients, Proteobacteria dominant profiles were associated with neutrophil activation markers, and Firmicutes dominant profiles were associated with raised blood eosinophil counts.^8^ Neutrophilic inflammation in COPD shows a predominance of *Haemophilus* and elevated levels of interleukin (IL)‐1β and tumor necrosis factor (TNF) in the sputum.^9^ In COPD patients, during exacerbations exist high biosynthesis of lipopolysaccharides; these molecules are components of the cell wall of gram‐negative bacteria^10^ and are induced locally in the lung release of neutrophils, production of IL‐1, and IL‐6. This inflammatory response contributes to transient airway obstruction.^11^

Reports regarding the involvement of the lung microbiome due to biomass‐burning exposure are very scarce; this smoke contains thousands of harmful substances, including particulate matter at most 10 (PM10) or 2.5 (PM2.5) microns in diameter.^12^ Smoke exposure is more prevalent in women in rural and suburban regions. In these cases, they use biomass fuel to cook and heat the home.^13^ Exposure for 30–40 years to biomass smoke is equivalent to 60,000 h or inhaling a total volume of 25 million/L of polluted indoor air during their lifetime.^14^ Indoor PM2.5 concentration in homes using biomass burning is higher than in non‐biomass‐burning (455 vs. 101 μg/m^3^). The same behavior is observed in 24 h CO concentrations (13.4 vs. 2.0 ppm).^15^

In the COPD Risk Cohort, the authors evaluated the influence of the environmental PM2.5 concentration on the lung microbiome for 14 days. The microbiome showed significant changes from the sixth day of exposure, especially in some Operational Taxonomic Units (OTUs). This change was maintained until the last monitored day.^16^ In a Malawian population, they observed that those exposed to high carbon particle levels had a greater abundance of potentially pathogenic bacteria (*Streptococcus* and *Neisseria*).^17^ Microbiome analysis provides an opportunity to develop novel prognostic markers for airway disease, enhance the definition of clinical phenotypes, acquire additional guidance to aid treatment selection, and increase the precision of indicators of a treatment effect.^18^ We aimed to characterize and compare the composition of the lung bacteriome and the cytokine profile in women without lung function affectation but exposed to environmental risk factors (tobacco smoking and exposure to smoke from biomass burning) for chronic lung diseases. We included women because, in low and middle‐income countries, they have a leading role in domestic, and the use of biomass has been a necessity for cooking or household heating; while men are at work or away from home.^19^

## 2 MATERIALS AND METHODS

### 2.1 Study population

We designed an observational, descriptive, and retrospective study, including women with biomass‐burning smoke exposure (BE, *n* = 11), recruited in rural and suburban populations by the Department of Research in Smoking and COPD (DITABE) of the Instituto Nacional de Enfermedades Respiratorias Ismael Cosío Villegas (INER) in Mexico City and current smoking women (TS, *n* = 10). The general inclusion criteria were women, age ≥40 years, with lung function test (forced expiratory volume in 1 second/forced vital capacity [FEV1/FVC]) values of ≥70%. The TS group had a history of ≥3 packs per year, and the BE group had a biomass smoke exposure index (BSEI) of ≥100 h per year. Both groups had exposure to corresponding environmental risk factors for ≥10 years. In addition, all participants had a physical examination by pulmonology specialists. The exclusion criteria were a record of respiratory diseases (asthma, COPD, cystic fibrosis, bronchiectasis, lung cancer, hypersensitivity pneumonitis, idiopathic pulmonary fibrosis, lung tuberculosis, etc.), lung transplant, cerebrovascular disease, psychiatric disorders, infectious disease, antibiotics, or immunosuppressive treatments in the previous 3 months, or evidence of acute upper respiratory symptoms for the preceding 4 weeks.

We applied a questionnaire to each participant about their place of birth, region of residence, ethnicity, sex, and employment status, including anthropometric characteristics, tobacco index (TI), or BSEI.

The lung function tests were performed by spirometry technicians considering the criteria established by the American Thoracic Society and the values calculated in the Mexican population.^20^ For postbronchodilator spirometry, a single‐dose salbutamol was administered using an inhaler and spacer.^21^

### 2.2 Sputum induction, processing, and bacterial DNA extraction

The women who agreed to participate voluntarily and signed an informed consent document were provided with a personal data protection certificate. The INER committee reviewed and approved the study on bioethics and research (protocol number B01‐17).

The women were required to stop smoking or leave biomass‐smoke exposure for at least 12 h before sample collection. Then, for sputum induction, the participant rinsed their mouth with water. Next, we administered sterile saline solution (7%) by nebulization for 5 min, followed by a 5‐min break. Participants performed this technique a maximum of three times.^22^ Finally, sputum was collected into sterile containers and transported to the laboratory.

In a biosafety cabinet, using a transfer pipette, saliva was eliminated. In a 1:1 relation, sterile saline solution (0.9%) was added to sputum using a sterile syringe and 20‐gauge needle to disaggregate sputum (up and down a minimum of 10 times); subsequently, we used a 22‐gauge needle until complete disintegration. The resulting sample was filtered (Falcon 70 µm Cell Strainer) and centrifuged at 3000 rpm at 20°C for 10 min. The supernatant was stored at −80°C. The cellular pellet was employed for bacterial DNA extraction. Bacterial DNA was purified using the ZymoBIOMICS™ DNA Miniprep Kit (Zymo Research Corp.), following the instructions for liquid samples (200 µL) as the supplier recommends. The DNA was quantitated utilizing the Qubit dsDNA High Sensitivity (HS) Assay Kit (Invitrogen) and assessed for integrity by electrophoresis on agarose gel. We included two saline samples to identify bacteria procedures of sources of contamination; these controls were treated in the same conditions that the sputum samples.

### 2.3 The 16Sr DNA metagenomic sequencing: Library preparation and sequencing

The libraries were prepared for targeted amplicon sequencing following the “16 Sr DNA Metagenomic Sequencing Library Preparation” guide (Part# 15044223 Rev. B, Illumina). We used the primer pair sequence for the V3 and V4 regions of the 16 S ribosomal RNA gene (16 Sr DNA), Nextera XT indices, and PhiX control. The sequencing was performed on the MiSeq platform using paired 300 bp reads and MiSeq v3 reagents. We employed MiSeq Control Software v 3.1.1.13 (Illumina) to generate the FASTQ files.

### 2.4 Bioinformatic analysis of the 16 Sr DNA sequences

We analyzed FASTQ files using RStudio,^23^ packages dada2,^24^ phyloseq,^25^ DESeq.2,^26^ ggplot2,^27^ microbiome,^28^ and a workflow for microbiome data analysis of Bioconductor (open‐source software for bioinformatics)^29^ was used. We employed the database of silva nr v138 and 97% similarity level to assign taxonomy.^30^ Community richness estimators included Chao1, Shannon, and Simpson indexes. The amplicon sequence variants (ASV) were obtained. Finally, the reads were normalized by cumulative‐sum scaling.^26^

### 2.5 Cytokines' immunoassay

According to the manufacturer's instructions, sputum supernatant cytokine levels were determined using a commercially available multiplex Human Cytokine Magnetic 10‐Plex Panel (Thermo Fisher Scientific). Proteins evaluated include IL‐1β, IL‐2, IL‐4, IL‐5, IL‐6, IL‐8, IL‐10, tumor necrosis factor (TNF)‐α, Interferon (IFN)‐γ, and granulocyte‐macrophage colony‐stimulating factor (GM‐CSF).

The multiplex assay was performed according to the manufacturer's instructions. Samples were homogenized and adjusted for further quantification in the Luminex LAB‐Scan 100 (Luminex Corp.) system. The cytokine concentrations were calculated using the standard curve. The xPONENT 3.1 software (Luminex Corp.) was used for data acquisition.

### 2.6 Statistical analysis

The analysis for anthropometric characteristics, TI or BSEI, spirometry data, and cytokine levels was performed using SPSS version 15 (SPSS software, IBM). For quantitative variables, we used a mean and standard deviation or medians and minimum and maxim values according to the distribution of the variables and used parametric or non‐parametric tests. Alpha diversity was also assessed using the Mann–Whitney *U* test. For the ASV differential abundance testing between groups, we employed the negative binomial generalized linear model (GLM) and obtained maximum likelihood estimates for an ASV log‐fold change between two study groups; the p‐value was adjusted by the false discovery rate (FDR) method.^31^ Furthermore, the p‐value of cytokine levels in sputum supernatant was obtained by the Mann–Whitney *U* test with a Bonferroni posthoc correction. A value of *p* < .05 was considered statistically significant. For the quantitative variables and abundance of bacteria, Spearman's correlation was made. The description of the results was carried out using the ggplot2^26^, ^27^ and corrplot^32^ packages in RStudio.^23^

## 3 RESULTS

### 3.1 Study population

The groups included in the study did not show statistically significant differences in age, body mass index, forced vital capacity (FVC), or expiratory volume in the first second (FEV1), as shown in Table 1. All the women in the TS group were from Mexico City. The TI was 16.7 packages per year, and they reported starting to smoke at 15 years old (12–25 years).

**Table 1:** Characteristics of the women included in the study.

Variable	BE (*n* = 11)	TS (*n* = 10)	*p*‐value^a^
Age (years)	60 (41–74)	53 (47–64)	.114
BMI (kg/m^2^)	28.1 (22.9–38.8)	27.3 (24.7–32.0)	.973
TI (package‐year)	–	16.7 (4–80)	–
BSEI (hours‐years)	216 (120–480)	–	–
Communities, *n* (%)			
Urban	0	10 (100)	–
Rural and suburban	5 (45.5)	0	
FVC %^b^	102 (86–134)	90 (81–124)	.223
FEV1%^b^	110 (90–155)	94 (82–131)	.099
FEV1/FVC %^b^	96 (82–114)	83 (71–88)	.001

*Note*: Showing medians (minimum–maxim values).

Abbreviations: BE, biomass‐smoke exposed women; BMI, body mass index; BSEI, biomass smoke exposure index; FEV1, forced expiratory volume in the first second; FVC, forced vital capacity; TI, tobacco index; TS, tobacco smoking women.

^a^ *p*‐value by the Mann–Whitney *U* test.

b Spirometry values are post‐bronchodilator.

Regarding the BE group, 45.5% of the women were born in Oaxaca state, the rest in the State of Mexico's rural and suburban areas, and reported having >210 h per year of exposure to smoke from biomass burning. The participants did not present any lung affection. The post‐bronchodilator FEV1/FVC was >70% in the study groups; however, the TS group had a lower FEV1/FVC ratio than the BE group (*p* = .001).

### 3.2 Composition of lung bacteriome

**Figure 1:**
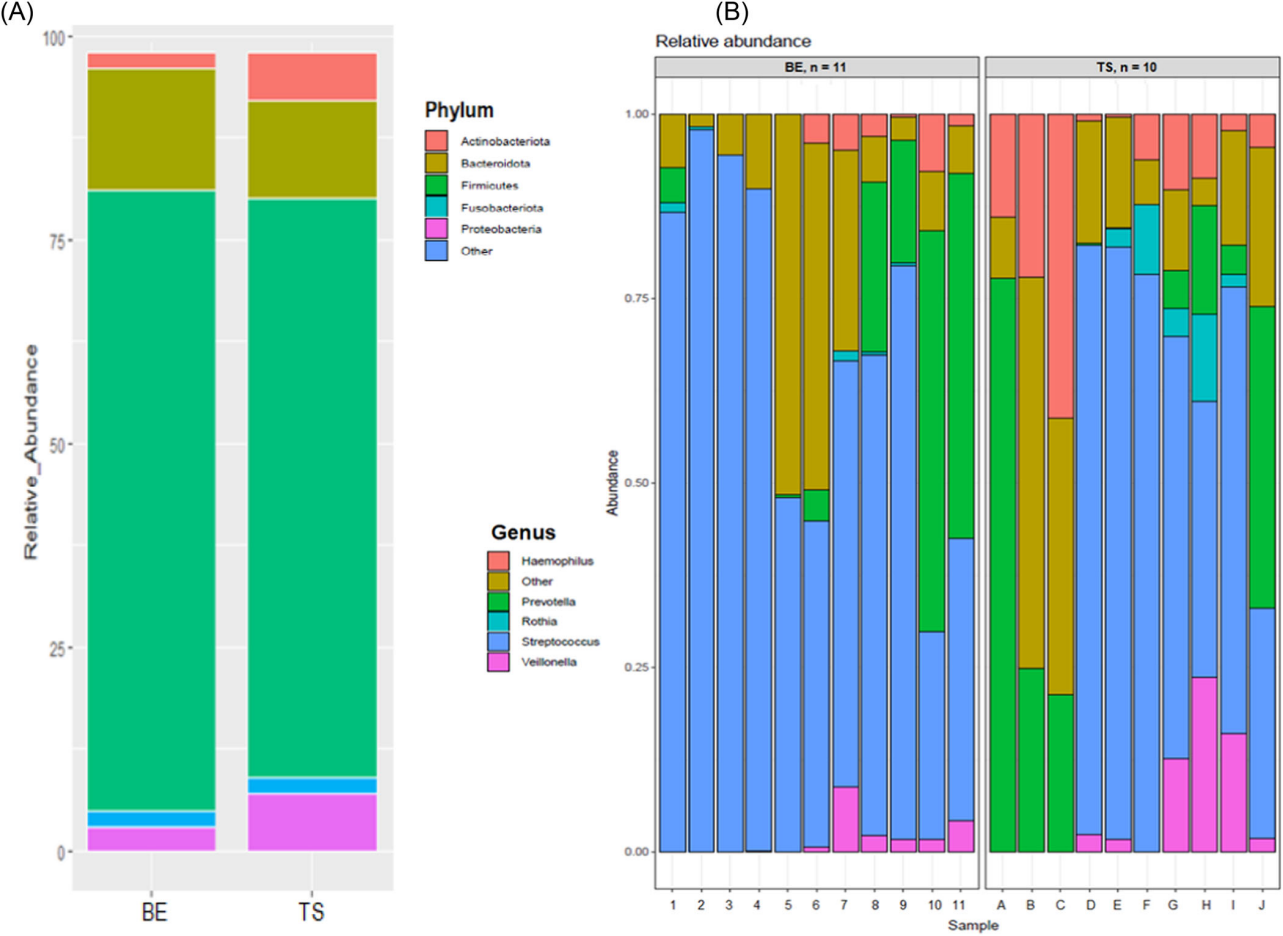
(A) Relative abundance in the study groups at the phylum level by the group. (B) relative abundance in the study BE versus TS by participants at the genus level. The numbers 1–11 correspond to the Biomass‐smoke exposed (BE) group, and A to J corresponds to tobacco smoking (TS).

We obtained >100,000 reads by sample. Comparing the bacterial composition in both study groups did not show a statistically significant difference in alpha diversity (Chao, *p* = .973; Shannon, *p* = .173; and Simpson, *p* = .282). The most abundant phyla were Actinobacteriota, Firmicutes, Bacteroidota, Proteobacteria, and Fusobacteriota. Proteobacteria was found in a higher proportion in the TS group concerning BE (*p* = .045); however, after the adjustment by FDR, this difference was not maintained (*p* = .288) (Figure 1A). The top five genera found were *Streptococcus*, *Veillonella*, *Haemophilus*, *Rothia*, and *Prevotella* (Figure 1B). However, there was no difference in relative abundance when groups were compared (Table 2). The data sets presented in this study (FASTQ file) can be found in online repositories (Sequence Read Archive, SRA; submission: SUB9326500).

**Table 2:** Differential abundance analysis for the top five bacteria between BE and TS.

Phylum	Class	Order	Family	Genus	*p*‐value*
Firmicutes	*Bacilli*	*Lactobacillales*	*Streptococcaceae*	*Streptococcus*	.135
	*Negativicutes*	*Veillonellales‐Selenomonadales*	*Veillonellaceae*	*Veillonella*	.455
Proteobacteria	*Gammaproteobacteria*	*Pasteurellales*	*Pasteurellaceae*	*Haemophilus*	.291
Actinobacteriota	*Actinobacteria*	*Micrococcales*	*Micrococcaceae*	*Rothia*	.246
Bacteroidota	*Bacteroidia*	*Bacteroidales*	*Prevotellaceae*	*Prevotella*	.511
Fusobacteriota	*Fusobacteriia*	*Fusobacteriales*	*Fusobacteriaceae*	*Fusobacterium*	.912
			*Leptotrichiaceae*	*Leptotrichia*	.926

Abbreviations: BE, biomass‐smoke exposed women; TS, tobacco smoking women.

* *p*‐value was adjusted by the false discovery rate (FDR) method.

**Figure 2:**
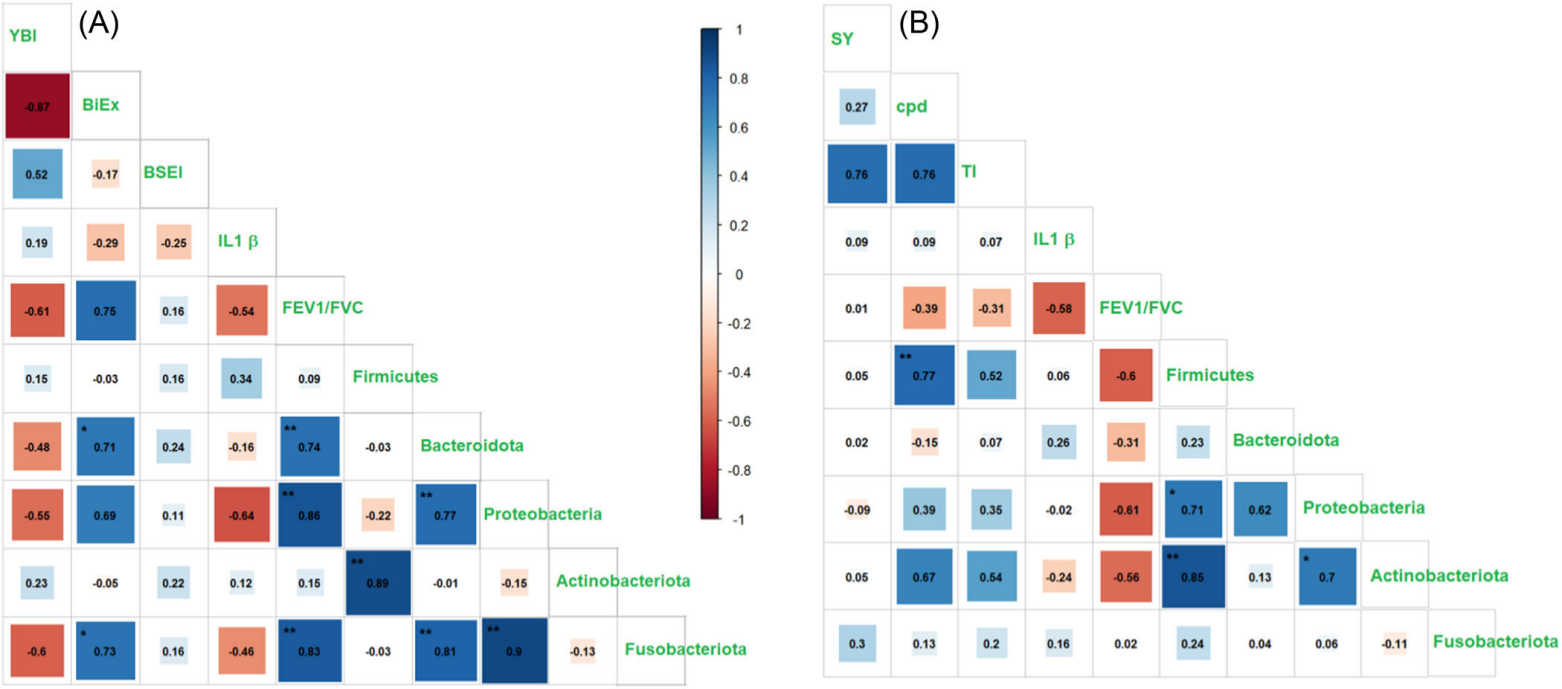
(A) Correlation analysis in women exposed to biomass‐burning smoke. (B) Correlation analysis in tobacco smoking group. *p < .05, **p < .01. BiEx, biomass‐burning smoke exposure in hours per day; BSEI, biomass smoke exposure index; cpd, cigarette per day; FEV1, Forced expiratory volume in the first second; FVC, forced vital capacity; SY, smoking years; TI, tobacco index; YBi, years to biomass‐burning smoke exposure.

In women exposed to biomass‐burning smoke, BSEI had a positive correlation with biomass‐burning smoke exposure in hours per day (BiEx) and abundance of Bacteroidota (*ρ* = 0.71, *p* = .014) and Fusobacteriota (*ρ* = 0.73, *p* = .011). FEV1/FVC had a positive correlation with an abundance of Bacteroidota, Proteobacteria, and Fusobacteria (*ρ* = 0.74, *p* = .009, *ρ* = 0.85, *p* = .001, and *ρ* = 0.83, *p* = .001, respectively). In the bacterial abundance analysis, we found that Firmicutes and Actinobacteria had a positive correlation (*ρ* = 0.88, *p* < .001). Bacteroidota had a positive correlation with Proteobacteria and Fusobacteriota (*p* = .001, ρ = 0.77 and *p* = .003, ρ = 0.80, respectively) and Proteobacteria had a positive correlation with Fusobacteriota (*p* < .001, *ρ* = 0.90) (Figure 2A).

In the analysis for the tobacco smoking group, we identified a positive correlation between cpd and Firmicutes (*p* = .009, *ρ* = 0.77). Firmicutes versus Proteobacteria and Actinobacteriota showed a positive correlation (*p* = .021, *ρ* = 0.71; *p* = .002, *ρ* = 0.85, respectively), and Proteobacteria and Actinobacteriota (*p* = .024, *ρ* = 0.70) (Figure 2B).

To assess whether the abundance of the obtained phylum correlated with the lung function of the patients, regardless of environmental exposure, we make Spearman's correlation analysis. Fusobacteriota showed a moderate positive correlation with FEV1% and FVC % postbronchodilator (*p* = .031, rho = 0.47 and *p* = .028, rho = 0.48 respectively) (Figure S1A,B).

### 3.3 Cytokines analysis

Regarding evaluated cytokines (Table 3), we found a higher concentration of IL‐1β in the TS group with respect to the BE group (248.6 vs. 177.9 pg/mL, *p* = .001) as well as GM‐CSF (142.2 vs. 140.6 pg/mL, *p* = .021) and IL‐2 (241.1 vs. 218.6 pg/mL, *p* = .340); however, with a Bonferroni posthoc correction, only IL‐1β maintained a significant association (*p* = .010).

**Table 3:** Sputum supernatant cytokine levels in both study groups.

Cytokine (pg/mL)	BE (*n* = 11)	TS (*n* = 10)	*p*‐value*
IL‐1β	177.9 (14.7–244.3)	248.6 (239.9–271.6)	**.010**
IL‐10	179.3 (137.5–184.7)	184.7 (178.2–186.9)	NS
IL‐6	264.8 (29.0–518.6)	107.6 (80.3–134.9)	NS
GM‐CSF	140.6 (43.3–98.9)	142.2 (140.6–158.3)	NS
IL‐5	362.8 (115.3–367.6)	364.9 (362.8–367.1)	NS
IFN‐γ	75.2 (60.7–111.83)	72.7 (65.9–79.4)	NS
TNF‐α	66.8 (29.4–82.8)	64.7 (63.1–69.7)	NS
IL‐2	218.6 (21.7–255.9)	241.1 (203.7–285.8)	NS
IL‐4	62.8 (62.8–715.6)	62.8 (62.8–84.6)	NS
IL‐8	1566.8 (894.5–4745.3)	1613.6 (1493.9–1655.1)	NS

*Note*: Showing medians (minimum–maxim values).

Abbreviations: BE, biomass‐smoke exposed women; NS, nonsignificative; TS, tobacco‐smoking women.

* *p*‐value was obtained by the Mann–Whitney *U* test with a Bonferroni post hoc correction.

## 4 DISCUSSION

The current study was carried out in women with exposure to known risk factors for the development of chronic lung diseases and without the presence of airflow limitation (FEV1/FVC ≥ 70).^33^ We found that the FEV1/FVC ratio of the TS group was lower compared to the BE group (83% vs. 96%). Previously, it has been reported that former smokers and low‐intensity current smokers (<5 cigarettes per day) have faster lung function deterioration compared to never‐smokers.^34^ Smoking is associated with a large range of alterations in systemic immune and inflammation marker levels.^35^ Patients with COPD secondary to tobacco smoking had more significant airflow obstruction,^36^ irritation, cough, phlegm, chronic bronchitis,^37^ higher FEV1 drop, and a higher degree of emphysema compared to COPD patients secondary to biomass smoke.^38^

We used sputum specimens to evaluate the microbiome in the women in the present study. The presence of oropharyngeal microbiota does not confuse the meaningful microbial signal in sputum, which is correlated with established indices of lung health.^2^ The human respiratory tract harbors a homogeneous microbiota that decreases bacterial biomass from the upper to the lower tract.^39^ The present study's alpha diversity does not show a statistically significant difference in both groups. Previously, a similar finding has been reported among patients with idiopathic interstitial pneumonia, sarcoidosis, and *Pneumocystis* pneumonia.^40^

The pulmonary bacterioma showed that the phylum Proteobacteria was statistically higher in the TS group concerning the BE group; however, this difference was not maintained after the Bonferroni correction (*p* = .288). The genera most abundant were *Streptococcus*, *Veillonella*, *Haemophilus*, *Rothia*, and *Prevotella*. We found no statistically significant difference between the BE and TS groups at the genus level.

Unfortunately, in this pilot study, we did not have healthy controls; however, we compared our data with previous reports and found that the relative average abundance of Firmicutes was higher (73.5%) in both groups compared to the lung microbiome of healthy subjects (27.8%–46.0%).^4^, ^41^ For Proteobacteria, the relative average abundance in our study (5%) was lower compared to that reported in the literature for volunteers without lung disease (16%–18.4%). Bacteroidota abundance in the healthy lung was similar to that reported in our study (16% vs. 13.5%). In healthy subjects, Actinobacteriota abundance has an extensive interval (1%–14%)^4^; in our study, we found that 4% of women were exposed to risk factors on average.

Exposure to smoke from biomass burning has been less explored; however, a biomass study on healthy people showed a higher abundance of *Streptococcus* and *Neisseria* are pathogenic bacteria.^17^ We found a high concentration of IL‐1β, IL‐2, and GM‐CSF in the TS group compared with BE; however, only IL‐1β maintained a significant difference after Bonferroni correction. IL‐1β is an essential mediator of chronic inflammation and the recruitment of neutrophils, eosinophils, and macrophages. The compounds in the smoke from cigarette smoking cause airway inflammation; an in vitro study of epithelial cells exposed to cigarette smoke extract showed that IL‐1β was expressed 9.5 times more than in unexposed cells.^42^ In the present communication, IL‐1β was 1.4 times higher in the TS group compared with BE. IL‐1β is a cytokine of innate immunity, involved in the initiation and maintenance of inflammation that participates in mucus hypersecretion and airway remodeling; it is overexpressed in epithelial cells of the small airway of COPD patients.^43^ Furthermore, it has been associated with an inflammatory mediator in exacerbations of bacterial etiology.^44^ The cytokine has been described to increase systemic inflammation, emphasizing its importance in exacerbating COPD and asthma.^45^

In this study, in women with biomass‐burning smoke exposure, no significant correlation between IL‐1β levels and the abundance of OTUs; however, high levels of BiEx increased the abundance of Bacteroidota and Fusobacteriota; Bacteroidota in the gut stimulate TLR2 but not TLR4; in the lung, this mechanism is unknown^46^; on the other hand, Fusobacteriota in cavity oral upregulated oncogenes, and promote tumorigenesis^47^; however in the lung, there are not reports.

In this study, we reported that the abundance of Proteobacteria has been correlated with FEV1/FVC in women exposed to biomass burning; the same observation exists in the abundance of Bacteroidota and Fusobacteriota. The phylum Proteobacteria has been associated with asthma^48^ and in COPD patients induces the production of inflammatory mediators in the lungs of COPD patients, contributing to tissue degradation in the lower airways and increased disease severity Proteobacteria.^49^ Interestingly, neutrophilic inflammation, increased pro‐inflammatory mediators (IL‐1β level increased significantly), and bacteria‐associated exacerbations with the predominance of Proteobacteria have been reported in a specific cluster with COPD patients that likely respond to antibiotics.^50^

In smoking women, the cpd was positively correlated whit Firmicutes abundance; previous studies showed that current smoking was strongly associated with the rate of FEV1 decline, and the most abundant phyla was Firmicutes.^51^

Alterations in the microbiota composition of the respiratory tract have been reported in the development of many chronic lung diseases (cystic fibrosis, COPD, and asthma) and are associated with more advanced conditions. Our pilot study is not exempt from limitations, such as the small sample size or the lack of a group of women without exposure to risk factors (healthy women). However, we included other reports in the literature to compare our results.

This is the first study that evaluates the bacterial composition in exposed women to known risk factors for lung diseases with normal lung function. We identified high levels of IL‐1β in the sputum of tobacco‐smoking women. In women with biomass‐burning smoke exposure exist a positive correlation between BiEx and abundance of Bacteroidota and Fusobacteriota. These changes in women exposed to environmental risk factors, despite not having lung function impairment, alter bacterial composition that contributes to inflammatory diseases such as COPD or asthma.

## 5 CONCLUSIONS

Compared to biomass‐burning smoke‐exposed women, current smokers have poor lung function and high levels of IL‐1β in sputum. Women with biomass‐burning smoke exposure present an increased abundance of Bacteroidota and Fusobacteriota.

## AUTHOR CONTRIBUTIONS

**Fernando Morales‐González**: Data curation; formal analysis; methodology; software. **Juan Alberto Lira‐Lucio**: Data curation; formal analysis; methodology; software. **Ramcés Falfán‐Valencia**: Conceptualization; funding acquisition; investigation; writing—original draft. **José Eduardo Márquez‐García**: Resources; validation. **Edgar Abarca‐Rojano**: Resources; validation; writing—review & editing. **Alejandra Ramírez‐Venegas**: Conceptualization; investigation; writing—original draft. **Raúl H Sansores**: Methodology; visualization. **Leonor García‐Gómez**: Resources; validation. **Andrea Hernández‐Pérez**: Resources; validation. **Gloria Pérez‐Rubio**: Conceptualization; formal analysis; project administration; resources; software; writing—original draft.

## CONFLICTS OF INTEREST STATEMENT

The authors declare no conflict of interest.

## INSTITUTIONAL REVIEW BOARD STATEMENT

The study was conducted according to the guidelines of the Declaration of Helsinki and approved by the INER committee on bioethics and research (protocol number B01‐17).

## INFORMED CONSENT STATEMENT

Informed consent was obtained from all subjects involved in the study.

## Supporting information

Supporting information.

## Data Availability

The data sets generated for this study can be found in the Sequence Read Archive (SRA). Submission: SUB9326500. Available in https://www.ncbi.nlm.nih.gov/bioproject/716362.
