# Supplementary material for: Characterization of the lung microbiome and inflammatory cytokine levels in women exposed to environmental risk factors: A pilot study
Source: Immun Inflamm Dis. 2023 Apr 17;11(4):e825. doi: 10.1002/iid3.825 (PMC10108684; doi:10.1002/iid3.825)
Supplement: Supplementary file 1 — Supporting information. [file IID3-11-e825-s001.pdf]

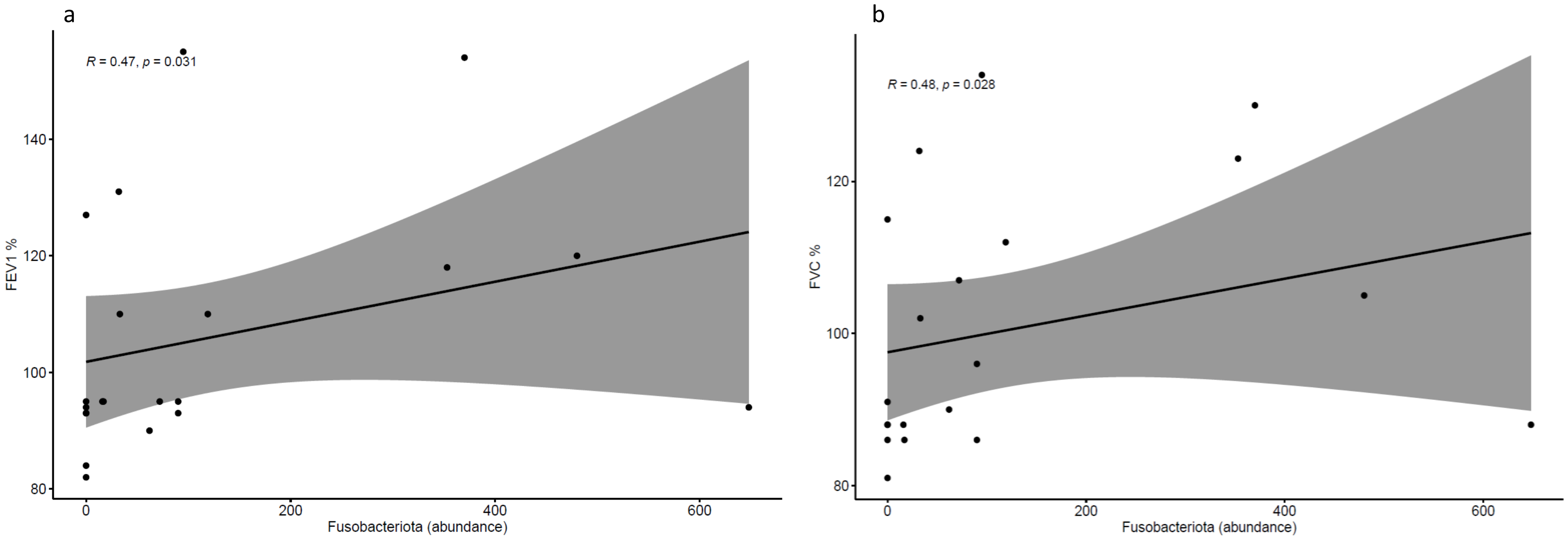

Supplementary figure 1. Spearman’s correlation analysis with all women included in this study. a) Fusobacteriota abundance showed a moderate positive correlation with FEV1 % ($p = 0.031$, rho = 0.47), and b) FVC % post-bronchodilator ($p = 0.028$, rho = 0.48).
